# Effect of fermented soy protein isolates containing collagen (Soylagen) on muscle atrophy: insight from network pharmacology analysis and experimental evidence

**DOI:** 10.3389/fphar.2026.1836847

**Published:** 2026-07-14

**Authors:** Young-Jin Choi, Nishala Erandi Wedamulla, Seok-Hee Kim, Kyoung-Min Rheu, Bae-Jin Lee, S. D. N. Kaushalya, Ji-Young Hwang, Eun-Kyung Kim

**Affiliations:** 1 Department of Food Science and Nutrition, Dong-A University, Busan, Republic of Korea; 2 Department of Health Sciences, Graduate School of Dong-A University, Busan, Republic of Korea; 3 Department of Food Science and Technology, Faculty of Animal Science and Export Agriculture, Uva Wellassa University, Badulla, Sri Lanka; 4 Smart Marine BioCenter, Marine Bioprocess Co., Ltd., Busan, Republic of Korea; 5 Department of Applied Bioscience, Graduate School of Dong-A University, Busan, Republic of Korea; 6 Department of Food Science and Technology, Dong-Eui University, Busan, Republic of Korea; 7 Nutritional Education Major, Graduate School of Education, Dong-A University, Busan, Republic of Korea; 8 Research and Development Center, Nutrinomics Lab. Co., Ltd., Busan, Republic of Korea

**Keywords:** collagen, fermented soy protein isolates, muscle atrophy, network pharmacology, PI3K/Akt/mTOR signaling pathway

## Abstract

**Introduction:**

Sarcopenia is an age-related condition marked by muscle atrophy, which leads to declined muscle strength and impaired physiological function, severely impacting the quality of life in older adults. Unfortunately, this condition poses a significant burden on both society and healthcare systems due to the lack of effective pharmaceutical treatments. As protein supplementation is crucial in managing muscle atrophy, recent studies have identified soy protein as a promising potential treatment. In light of this, the current study aims to investigate the effects of fermented soy protein isolates containing collagen (Soylagen) on muscle atrophy through both in vitro and in vivo studies, along with network pharmacology analysis.

**Method:**

Network pharmacology was conducted to investigate the potential mechanisms underlying the effects of Soylagen on muscle atrophy. In addition, *in vitro* and *in vivo* studies were performed to evaluate its anti-muscle atrophy effects and associated molecular pathways.

**Results and discussion:**

Network analysis suggests that the beneficial effects of fermented Soylagen on muscle atrophy are primarily linked to the biosynthesis of branched-chain amino acids, L-valine, glutathione, amino acids, and the MHC class I peptide loading complex. *in vitro* and *in vivo* studies demonstrated that fermented Soylagen significantly upregulated the protein expression of PI3K, p-Akt, and p-mTOR, indicating that its effects on muscle atrophy may be mediated through the PI3K/Akt/mTOR pathway. In conclusion, fermented Soylagen shows potential as an effective natural remedy for muscle atrophy.

## 1 Introduction

The rapidly growing rate of the elderly population has become a major challenge the world is facing today, with reports indicating that one out of six people will be 65 years or older globally by 2050 (Jee, 2024). With this prediction, the global attention towards age-related disease has gradually increased. Sarcopenia generally featured in older adults and characterized by muscle atrophy which leads to progressive loss of muscles, strength and function (Zhu et al., 2023). This may potentially cause several conditions such as falls, fractures, disabilities and even death (Kim et al., 2024). Studies have proven that sarcopenia has affected the quality of life of older adults worldwide, accounting for 10%–16% (Meng et al., 2024). Apart from the adverse effects of sarcopenia on the quality of life of the elderly population, it also burdens the entire medical system while increasing economic issues for families and societies. Prevalence rates of sarcopenia vary gradually across the world, affecting around 5%–13% of the elderly population (>62 years old) with low muscle mass and weakened skeletal muscle function, while increasing the risk of injury in those aged 80 and above up to 50% (Nishikawa et al., 2021). South Korea has reported a prevalence of 13.1% in the elderly aged 60 years and above as revealed by the epidemiological studies. Meanwhile, Japan reported a prevalence of 9.9% (Meng et al., 2024).

Pathogenesis of sarcopenia is not fully elucidated, and several factors were identified as the major causes of sarcopenia where imbalance between the synthesis and degradation of skeletal muscle protein plays a pivotal role. Moreover, malnutrition, sedentary lifestyle, hormonal changes, myokines, inflammation, oxidative stress and mitochondrial dysfunction also equally contributed to sarcopenia (Liao et al., 2019). In regards to muscle atrophy, muscle quantity and quality are primarily affected by proteins, which are mainly supplied through food. However, the majority of the elderly fail to reach the recommended dietary allowance of protein, aggravating muscle atrophy, as protein consumption plays a key role in stimulating muscle protein synthesis (Zhu et al., 2023). Subsequently, high-quality protein supplementation and strength training have been identified as promising measures for the prevention of sarcopenia (Frias et al., 2008).

Soy foods are recognized as promising sources of protein, vitamins, minerals, and fiber. However, their acceptance is challenging due to allergic reactions. Recent studies have identified fermentation as a promising technique to improve the nutritional and functional properties of soy products where low molecular weight proteins, polypeptides, oligopeptides and isoflavone are enhanced through fermentation process (Frias et al., 2008; Das et al., 2023). Further, recent studies have also confirmed that lactic acid bacteria fermentation can modulate the characteristics and functions of protein while increasing its digestibility (Jayachandran and Xu, 2019). As evidenced by previous studies, lactic acid bacteria produce proteolytic enzymes that hydrolyze proteins into peptides and free amino acids. This process can also generate functional peptides apart from the degradation of soy allergens (Xu et al., 2017). The studies also reported that the fermentation of soy products with different microorganisms contributed to an increase in antioxidant activity, Gamma-aminobutyric acid (GABA), and vitamin B_2_ and B_12_ (Zdzieblik et al., 2015).

Collagen peptides are also identified as a promising treatment option for sarcopenia where collagen-supplemented group exhibits an increase in muscle strength in older subjects with muscle atrophy. On the other hand, collagen is known to contain high amounts of arginine and glycine (Hays et al., 2009). Furthermore, the study also confirmed that consuming collagen peptides was more effective than whey protein isolates (WPI) in maintaining nitrogen balance and body weight when following a low-protein diet (McKendry et al., 2024). Generally, a lack of physical activity, combined with reduced masticatory and digestive functions in the elderly, leads to insufficient resistance exercise and inadequate consumption of high-quality protein. In this context, collagen plays a crucial role in strengthening muscles and regulating and regenerating muscle tissue (Kim et al., 2024). Consequently, collagen supplementation can be offered alternative for older individuals with muscle atrophy.

Number of studies have investigated the effect of fermented proteins and collagen supplementation on muscle atrophy and sarcopenia. However, the effect of fermented soy protein isolates in combination with collagen on sarcopenia has not been investigated in the previous studies to the best of our knowledge. Considering this research gap, the current study aimed to investigate the effect of fermented soy protein isolates containing collagen on sarcopenia.

## 2 Materials and methods

### 2.1 Materials and reagents

Fermented soy protein isolates containing collagen used in the current study commercially named as Soylagen and was kindly donated by the Marine Bioprocess Co., Ltd. (Busan, Republic of Korea) and soy protein isolates were obtained from the Shandong Wonderful Industrial group co. Ltd, Shandong, China. Whey protein isolates (WPI; 99.9%) were purchased from Sachsenmilch Leppersdorf GmbH (Leppersdorf, Germany). Dulbecco’s modified eagle medium (DMEM) and fetal bovine serum (FBS) were purchased from Welgene (Daegu, Korea). Penicillin/streptomycin (P/S) was obtained from Gibco (Grand Island, NY, United States). 3-(4,5-Dimethylthiazol-2-yl)-2,5-dipheny tetrazolium bromide (MTT) was procured from Invitrogen (Waltham, MA, United States). Alanine aminotransferase (ALT) (ab105134) and aspartate aminotransferase (AST) (ab105135) were procured from Abcam (Cambridge, England). Antibody against Myogenin (MyoG), myoblast determination protein 1 (MyoD), Myosin Heavy Chain (MYH), Glyceraldehyde-3-phosphate dehydrogenase (GAPDH), hosphoinositide 3-kinase (PI3K), phosphorylated protein kinase B (p-Akt) and Akt were purchased from Santa Cruz Biotechnology (Dallas, TX, United States). Antibodies against phosphorylated mammalian target of rapamycin (p-mTOR) and mTOR were procured from Cell Signaling Technology (Danvers, MA, United States).

### 2.2 Preparation of fermented soy protein isolates containing collagen

Soy protein isolates were hydrolyzed at 60 °C ± 5 °C for 4 h with alcalase (Novozymes, Bagsværd, Denmark). Production of Soylagen was carried out through two consecutive fermentation processes using *L. brevis* BJ20 (accession No. KCTC 11377BP) and *L. plantarum* BJ21 (accession No. KCTC 18911P). The seed medium, composed of 3% yeast extract (Choheung, Ansan, Republic of Korea), 1% glucose (q1, Seoul, Republic of Korea), and 96% water, was sterilized at 121 °C for 15 min. After sterilization, it was inoculated with 0.02% *Lactobacillus brevis* BJ20% and 0.02% *Lactobacillus plantarum BJ21*, and then separately incubated at 37 °C for 24 h. In the first fermentation, 10% (v/v) of the *L. brevis* BJ20 cultured seed medium was introduced into a fermentation medium containing 3% yeast extract, 1% glucose, 29% collagen (Geltech Co., Ltd., Busan, Republic of Korea), 1% monosodium glutamate (MSG) (CJ, Seoul, Korea), and 47% water, and the mixture was fermented at 37 °C for 24 h. Afterward, 10% (v/v) of the *L. plantarum* BJ21 cultured seed medium was added, and fermentation continued at 37 °C for an additional 24 h. The resulting fermentation mixture was filtered using a filter press (Daehanfilter, Chungju, Korea), sterilized, and spray-dried (Naturalendo Tech, Kyonggido, Republic of Korea) to produce Soylagen powder samples.

### 2.3 Network pharmacology analysis

#### 2.3.1 Exploring the potential targets of Soylagen and sarcopenia

The keyword “sarcopenia” was used to search for the potential targets related to the disease of interest using Comparative Toxicogenomics Database (CTD; https://ctdbase.org/, accessed on 25 December 2024). Then the potential targets of Soylagen were predicted using the STITCH database (http://stitch.embl.de/, accessed on 25 December 2024). Hydroxyproline, glycine, hydroxylysine, aspartic acid, glutamic acid, arginine, alanine, proline and lysine were identified as the primary compounds present in fermented soy protein isolates and collagen (Das et al., 2023; Kim et al., 2018). The medium confidence cut-off of 0.400 was applied, restricting the species to *Homo sapiens* only. The active compound-target interaction network of Soylagen was visualized with the Cytoscape software (version 3.10.1).

#### 2.3.2 Construction of protein-protein interactions (PPI) network

The online tool Venny 2.0.2 (https://bioinfogp.cnb.csic.es/tools/venny/index2.0.2.html, accessed on 25 December 2024) was used to identify the targets of Soylagen that overlap with the screened disease targets. After identifying the potential therapeutic targets of Soylagen against sarcopenia, these targets were imported into the STRING database (https://string-db.org/, accessed on 25 December 2024) to construct a PPI network. A confidence value greater than 0.4 was applied. The Cytoscape software (version 3.10.1) was employed to visualize the PPI network. The top ten hub genes were identified using CytoHubba, a plug-in for the Cytoscape software and ranked by betweenness.

#### 2.3.3 Gene ontology (GO) and Kyoto encyclopedia of genes and genomes (KEGG) pathway enrichment analyses

The GO and KEGG enrichment analysis was carried out using the Database for Annotation, Visualization, and Integrated Discovery (DAVID: https://david.ncifcrf.gov/tools.jsp, Accessed on 25 December 2024). The results of the GO analysis were presented in the biological process (BP), cellular components (CC), and molecular function (MF) categories, with a significance threshold of p < 0.05. Additionally, the results were visualized in the form of a histogram using SRplot (https://www.bioinformatics.com.cn/en, accessed on 25 December 2024). The top 20 pathways were selected for KEGG enrichment analysis and visualized from bubble plot using SRplot.

### 2.4 Cell culture and treatment

#### 2.4.1 Cell culture

The murine myoblast cell line C2C12 cells were purchased from the American Type Culture Collection (ATCC; Manassas, VA, United States). The cells were cultured in DMEM supplemented with 10% FBS and 1% P/S at 37 °C in a humidified environment with 5% CO_2_. The cells were sub-cultured when they reached 80% confluence.

#### 2.4.2 Cell viability

C2C12 cells were seeded into 96-well plate at a density of 5 × 10^3^ cells/well and incubated at 37 °C under 5% CO_2_. After 24 h incubation period, the cells were treated with different concentrations (10–500 μg/mL) of fermented Soylagen for 24 h at 37 °C. Following the 24 h treatment, the wells were treated with MTT (5 mg/mL) for 4 h. Then, the medium was removed, and dimethyl sulfoxide (100 µL) was added to each well to measure the absorbance at 540 nm. Cell viability was calculated as a percentage relative to that of untreated well.

#### 2.4.3 Differentiation of C2C12 cells

First, C2C12 cells were seeded in six well plates at a density of 1 х 105 cells/well and cultured in growth medium until reaching 85%–95% confluence. To induce myogenic differentiation, the growth medium was replaced with differentiation medium consisting of DMEM supplemented with 2% horse serum (HS). For treatment groups, cells were exposed to Soylagen, fermented Soylagen, or WPI at concentrations of 50 and 100 μg/mL at the onset of differentiation. The differentiation medium containing the respective treatment was refreshed every 28 h, and cells were maintained under these conditions for 6 days. At the end of the differentiation period, cells were washed with phosphate-buffered saline (PBS), fixed with 10% buffered formalin, and subjected to hematoxylin and eosin (H&E) staining. Morphological changes and myotube formation were observed under a light microscope to evaluate myogenic differentiation.

#### 2.4.4 Reverse transcription quantitative PCR

Following treatment, total RNA was extracted from C2C12 cells using Trizol, according to the manufacturer’s instructions. cDNA synthesis was carried out using AccuPower RT PreMix (Bio-Rad, Hercules, CA, United States). Quantitative PCR was performed for, MyoD, MyoG, MyH1 and GAPDH using a MIC qPCR cycler (Bio Molecular Systems, Upper Coomera, Australia). The ΔCt value was calculated as the difference between the target gene Ct and GAPDH Ct, and gene expression was expressed as 2^−ΔΔCt^ using the Ct values provided by the micPCR software.

#### 2.4.5 Western blot analysis

Following the treatments the cells were harvested with RIPA lysis buffer and centrifuged at 13,000 RPM/min (15,928 g) for 20 min at 4 °C until the supernatant was separated. Protein concentrations were estimated using Bicinchoninic acid (BCA) assays, and proteins were separated by 12% SDS-PAGE (90 min at 120 V). Transfer of the separated proteins to a nitrocellulose membrane was carried out using a Mini Trans-Blot cell (Bio-Rad, CA, United States). The membranes were then blocked with 5% skim milk for 1 h at room temperature (25 °C ± 2 °C). Following blocking, the membranes were incubated overnight at 4 °C with primary antibodies, all diluted at 1:1,000. After three washes with Tris-buffered saline + Tween 20 (TBST), the membranes were incubated for 1 h at room temperature with the corresponding secondary antibodies. The membranes were washed three more times with TBST, and chemiluminescence was developed using a horseradish peroxidase substrate (Advansta Inc., San Jose, CA, United States). Finally, the chemiluminescent signals were captured using an Azure c300 imaging system (Azure Biosystems, Dublin, CA, United States).

#### 2.4.6 Immunofluorescence

C2C12 cells were treated with fermented Soylagen (50 and 100 μg/mL) and WPI (100 μg/mL) as explained in the above section. The cells were seeded in 8-well slide chamber (SPL Life Science Co., Seoul, Republic of Korea). Following the treatments, the cells were fixed in ice-cold methanol, followed by permeabilization with 0.1% Triton X-100 for 15 min. Next, the cells were blocked with 5% normal goat serum, and the slides were incubated overnight at 4 °C with α-tubulin (1:300). Afterward, they were incubated with the corresponding goat anti-mouse IgG for 1 h. Mounting medium containing 4′,6-diamidino-2-phenylindole (DAPI) was added to the slides, and coverslips were mounted. Images were captured using a Zeiss 700 confocal microscope (Oberkochen, Germany).

### 2.5 *In vivo* experiment

#### 2.5.1 *In vivo* experimental design

Male C57BL/6N (8 weeks old; Nara Biotech Co. Ltd., Pyeongtaek, Korea) were used to investigate the effect of fermented Soylagen on muscle atrophy. All animals’ procedures were approved by the Institutional Animal Care and Use Committee of Dong-A University (DAIACUC-23-58). Mice were housed in pathogen-free conditions in clear plastic cages with aspen bedding, maintained at 20 °C–21 °C, 40%–45% relative humidity, and a 12 h light/dark cycle, with *ad libitum* access to standard diet and water. Following a 1-week acclimatization period, animals were randomly assigned to five groups (n = 6 per group):

1. Control (CON)
2. Dexamethasone-treated (DEX)
3. DEX + fermented Soylagen (400 mg/kg; DEX + S_L)
4. DEX + fermented Soylagen (800 mg/kg; DEX + S_H)
5. DEX + WPI (800 mg/kg; DEX + WPI)

**FIGURE 1:**
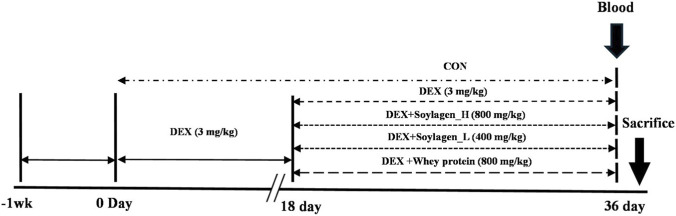
*In vivo* experiment design. Following the 1 week of acclimatization period, dexamethasone (DEX) was administered intraperitoneally for 36 days to induce skeletal muscle degeneration. After 18 days of DEX treatment, Soylagen and whey protein isolates (WPI) were oral administered for 18 days with the treatment of DEX. The animals in the control group (CON) did not receive any treatment.

Muscle atrophy was induced by intraperitoneal injection of DEX (3 mg/kg/day) for 36 days. To evaluate the therapeutic effects of fermented Soylagen and WPI, these treatments were administered orally via oral gavage once daily during the last 18 days of the DEX treatment period (Day 19–36). The control group received no treatment. At the end of the experimental period (Day 36), mice were euthanized, and skeletal muscle tissues including soleus (S), gastrocnemius (GA), and tibialis anterior (TA) were collected. The GA muscle was frozen and stored at −80 °C for Western blot analysis, while the portion was fixed in 10% formaldehyde for histological evaluation. Blood samples were collected via cardiac puncture, and serum was separated and stored at −80 °C for further analysis. The overall experimental design is illustrated in Figure 1.

#### 2.5.2 Grip strength

Grip strengths were measured after the 36 days of treatment period using a digital grip strength meter (BIO-GS4, BIOSEB, France). Mice were placed on a net connected to a force sensor, and their tail was pulled back from the horizontal plane using a constant force just before the mice fell off the grid. Muscle strength was measured in grams (g) with three replicate measurements.

#### 2.5.3 Swimming test

Forced swimming was conducted in a glass cylinder (diameter, 10 cm; height, 30 cm) containing warm water (temperature, 28 °C ± 1 °C) to a 15-cm depth for 2 min according to the methos described in previous study (dos Santos et al., 2023).

#### 2.5.4 Serum AST and ALT levels

Serum was separated by centrifuging mouse blood samples at 4 °C and 159,28× g for 20 min. The separated serum was stored at −80 °C until further analysis. Serum AST and ALT levels were determined using transaminase assay kits, following the manufacturer’s instructions.

#### 2.5.5 Western blot analysis

Gastrocnemius muscle tissues were homogenized and lysed with RIPA lysis buffer, then centrifuged at 13,000 rpm (15,928 g) for 20 min at 4 °C to separate the supernatant. After determining the protein concentration, the proteins were separated, transferred, and detected as described in Section 2.4.5.

#### 2.5.6 H&E staining

The gastrocnemius muscle was fixed in 10% formaldehyde, embedded in paraffin, and sectioned to a thickness of 4 μm using a Leica microtome (RM 2135, Wetzlar, Germany). The sections were deparaffinized, hydrated, and stained with hematoxylin for 5 min, followed by a 5-min wash in water. Eosin staining was performed for 30 s. After dehydration, the sections were cleared in xylene and mounted. Histological analysis was conducted using a Leica DMi1 Research Inverted Phase microscope at ×200 magnification.

#### 2.5.7 Immunohistochemical analysis

Antigen retrieval was carried out on sections (4 μm) that had been deparaffinized and rehydrated by immersing them in 0.01 M citrate buffer (pH 6.0). The slides were then subjected to microwave heating for 10 min, followed by a 10-min incubation at room temperature and a rinse with tap water. After blocking with 5% normal goat serum, the sections were incubated overnight at 4 °C with MyoD (diluted 1:300). This was followed by incubation with the appropriate goat anti-mouse IgG, and FSD™ 488-conjugated goat anti-mouse IgG for 1 hour. The nuclei were stained with 4′,6-diamidino-2-phenylindole (DAPI). Finally, the slides were mounted with a mounting medium and cover slip. Images were captured using a Zeiss 700 confocal microscope (Oberkochen, Germany).

### 2.6 Statistical analysis

The data are presented as mean ± standard deviation (SD) of at least three replicates and were analyzed using GraphPad Prism software (Version 8.4; GraphPad Software, Inc., United States). Data normality was assessed using the Shapiro–Wilk test, and all datasets were found to follow a normal distribution (p > 0.05). Accordingly, differences among multiple groups were analyzed using one-way analysis of variance (ANOVA) followed by Scheffé’s *post hoc* multiple comparison test. To further validate the robustness of the results, a non-parametric Kruskal–Wallis test was also performed. A p-value < 0.05 was considered statistically significant.

## 3 Results

### 3.1 Network pharmacology analysis

**FIGURE 2:**
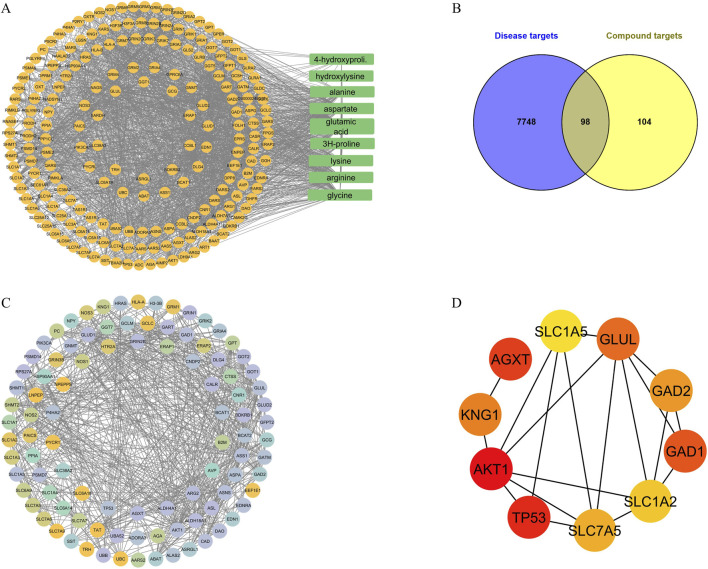
Identification of core targets using network pharmacology analysis. **(A)** The compound-target protein network constructed by Cytoscape. **(B)** Venn diagram screening 98 core targets related to sarcopenia. **(C)** The PPI network of 98 core targets constructed by Cytoscape. Varied sizes and color intensities of the nodes denoted the STRING score. **(D)** Top 10 hub genes screened from betweenness algorithm of the CytoHubba plugin of Cytoscape. Varied color intensities denoted the rank, where red represents the highest rank while yellow represents the lowest.

The network pharmacology analysis identified a total of 202 targets for Soylagen with medium confidence (0.4), using the STITCH database. Supplementary Table S1 displays the corresponding targets related to the major constituents of Soylagen. Based on these targets, a compound-target protein network was constructed using Cytoscape and is illustrated in Figure 2A. A total of 7,846 differentially expressed disease genes were identified using the Comparative Toxicogenomics Database with the keyword “sarcopenia”. Supplementary Table S2 displays the respective diiferentially expressed genes related to sarcopenia. Using the compound and disease targets, 98 core targets were screened via a Venn diagram (Figure 2B). The complex interactions among these 98 core targets are depicted in the PPI network, constructed with Cytoscape (Figure 2C). The varying color intensities in the PPI network represent the STRING interaction score. The PPI network consists of 98 nodes and 736 edges. The CytoHubba plugin of Cytoscape was used to screen the top ten hub genes, which are displayed in Figure 2D. These top ten hub genes were selected based on betweenness centrality. AKT Serine/threonine kinase 1 (AKT1), TP53, alanine-glyoxylate aminotransferase (AGXT), Glutamate decarboxylase 1 (GAD1), and Glutamate-ammonia ligase (GLUL) were identified as key hub genes associated with Soylagen in sarcopenia.

**FIGURE 3:**
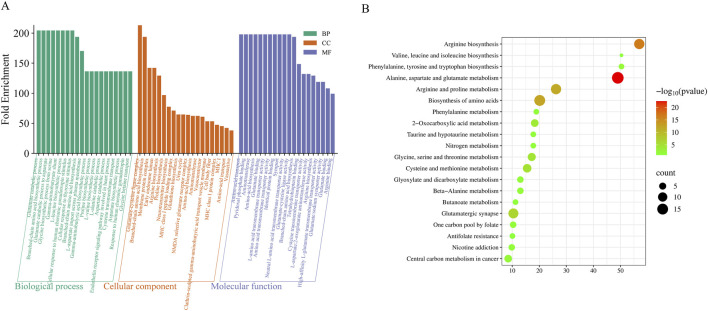
Enrichment analysis illustrating the effects of Soylagen on muscle atrophy **(A)** GO enrichments analysis of 98 core targets of Soylagen. **(B)** KEGG enrichments analysis of 98 core targets of Soylagen.

The results of the GO and KEGG enrichment analysis from the network pharmacology study are shown in Figures 3A,B, respectively. As shown in Figure 3A, the GO analysis of the potential targets of Soylagen in sarcopenia primarily indicates its effects through biological processes involved in protein biosynthesis, with branched-chain amino acid biosynthesis and L-valine biosynthetic processes identified as key biological processes. Furthermore, the GO enrichment analysis also identified the MHC class I peptide loading complex, glutathione biosynthesis, and amino acid biosynthesis as major cellular components. Similarly, the KEGG pathway analysis identified the biosynthesis of amino acids, arginine biosynthesis, and the biosynthesis of valine, leucine, and isoleucine as key KEGG pathway terms associated with the core targets of Soylagen in sarcopenia (Figure 3B).

The present study quantified the levels of key amino acids in Soylagen to validate the predicted metabolic pathways identified through network analysis (Supplementary Figure S1). Consistent with the findings of the network analysis, alanine, leucine, valine, and isoleucine (Supplementary Table S1) were identified as the predominant amino acids in Soylagen.

### 3.2 Effect of Soylagen and fermented Soylagen on myoblast differentiation

**FIGURE 4:**
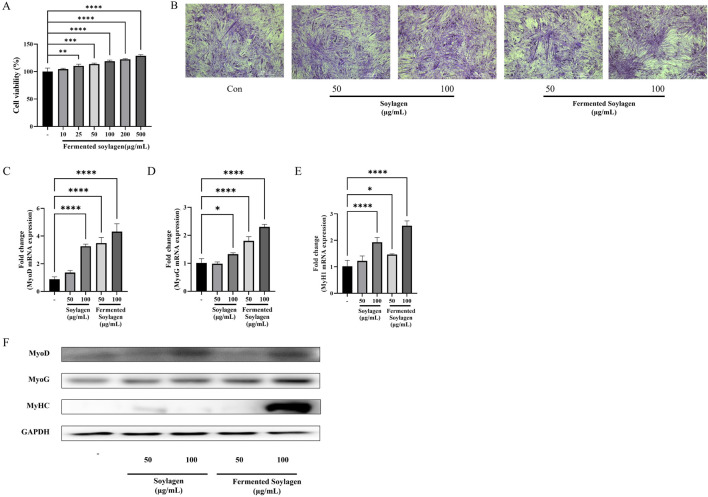
Effect of fermented Soylagen on myogenic differentiation in C2C12 cells. **(A)** The cell viability of C2C12 myoblast cells with different concentrations of fermented Soylagen. **(B)** Representative images of C2C12 cells differentiated for 6 days with the treatment of Soylagen and fermented Soylagen. Relative mRNA expression levels of **(C)** MyoD **(D)** MyoG and **(E)** MyH1 in C2C12 cells treated with Soylagen and fermented Soylagen. **(F)** Representative Western blot images of protein expression levels of MyoD, MyoG and MyHC in C2C12 cells. Data are expressed as the mean ± SD. *p < 0.05, **p < 0.01, ***p < 0.001 and ****p < 0.0001 compared with control group.

The current study initially assessed the effect of fermented Soylagen on the cell viability of C2C12 cells using the MTT assay, with the results shown in Figure 4A. The data indicated that fermented Soylagen did not affect cell viability within the tested range of 10–500 μg/mL. To evaluate the impact of Soylagen and fermented Soylagen on myotube differentiation, C2C12 myoblasts were induced to differentiate by switching to a differentiation medium containing 2% HS and were treated with Soylagen and fermented Soylagen for 6 days. As illustrated in Figure 4B, both Soylagen and fermented Soylagen promoted myotube differentiation in a dose-dependent manner, with fermented Soylagen showing a more pronounced effect on myotube formation, as evidenced by the increased length and diameter of the myotubes. To further confirm the effect of Soylagen and fermented Soylagen on myoblast differentiation, the gene expression levels of MyoD, MyoG, and Myosin Heavy Chain 1 (MyH1) were measured and shown in Figures 4C–E. As shown in Figure 4C, the expression level of MyoD significantly (p < 0.05) increased with the treatment of both Soylagen and fermented Soylagen in a concentration-dependent manner, with fermented Soylagen demonstrating a more pronounced effect. A similar pattern was observed in the expression levels of MyoG and MyH1, further confirming the enhanced effect of fermented Soylagen on myoblast differentiation. To further confirm the effect of Soylagen on C2C12 myoblast differentiation, the current study also evaluated the protein expression levels and the results presented in Figure 4F. The protein expression of MyoD, MyoG, and MyHC exhibited a similar trend, with increased levels following treatment with Soylagen compared to the control. Consistent with the gene expression results, the protein expression levels of MyoG and MyHC in cells treated with fermented Soylagen were higher than those in cells treated with raw Soylagen.

### 3.3 Effect of fermented Soylagen and WPI on myoblast differentiation

**FIGURE 5:**
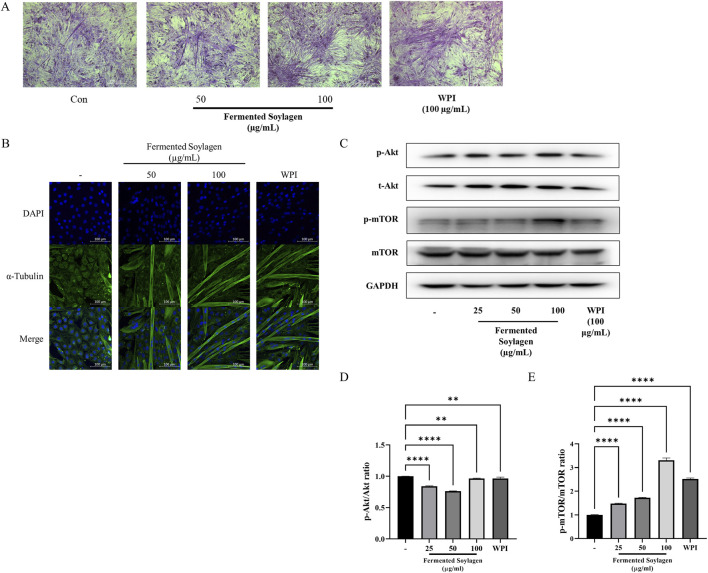
Effect of fermented Soylagen and whey protein isolates (WPI) on myogenic differentiation in C2C12 cells. **(A)** Representative images of C2C12 cells differentiated for 6 days with the treatment of fermented Soylagen and WPI. **(B)** Immunofluorescence staining of α-tubulin (green) and DAPI (blue) in C2C12 myotubes. C2C12 myoblasts were treated with or without the indicated concentrations of fermented Soylagen and WPI (100 μg/mL) in differential medium for 6 days. **(C)** Representative Western blot images of protein expression levels of p-Akt, Akt, p-mTOR and mTOR. Histograms for protein expressions of **(D)** p-Akt/Akt and **(E)** p-mTOR/mTOR. Data are expressed as the mean ± SD. ***p < 0.001 and ****p < 0.0001 compared with control group.

The current study aimed to compare the effects of fermented Soylagen and WPI on myoblast differentiation, as WPI is well-known for its beneficial effects on muscle mass and strength (dos Santos et al., 2023; Boutry-Regard et al., 2020; Cereda et al., 2019). To evaluate the impact of fermented Soylagen and WPI on myoblast differentiation, C2C12 cells were treated with 50 and 100 μg/mL concentrations of fermented Soylagen, and 100 μg/mL of WPI, after the medium was changed to differentiation medium. As shown in Figure 5A, treatment with both high concentrations of fermented Soylagen and WPI resulted in similar outcomes, with a marked increase in the length and diameter of the myotubes compared to the control group. The current study further confirms the effects of fermented Soylagen and WPI on myoblast differentiation through immunofluorescence using an α-tubulin antibody, with the results shown in Figure 5B. As illustrated in the figure, the average number of DAPI-stained nuclei localized in α-tubulin-positive myotubes gradually increased with the treatment of fermented Soylagen and WPI compared to the control. Moreover, this effect was concentration-dependent, with the 100 μg/mL treatment of fermented Soylagen being more effective than the 50 μg/mL treatment. Therefore, at higher concentration, both fermented Soylagen and WPI exhibited similar effects. To further confirm the effect of fermented Soylagen and WPI on myoblast differentiation, the current study determined the protein expressions levels of p-Akt and p-mTOR and results shown in Figures 5C–E. A concentration-dependent increase in the p-mTOR/mTOR ratio was observed (Figure 5E). However, no significant change in the p-Akt/Akt ratio was detected after treatment with either fermented Soylagen or WPI (Figure 5D).

### 3.4 Effect of fermented Soylagen on skeletal muscle mass and muscle performance in mice

**FIGURE 6:**
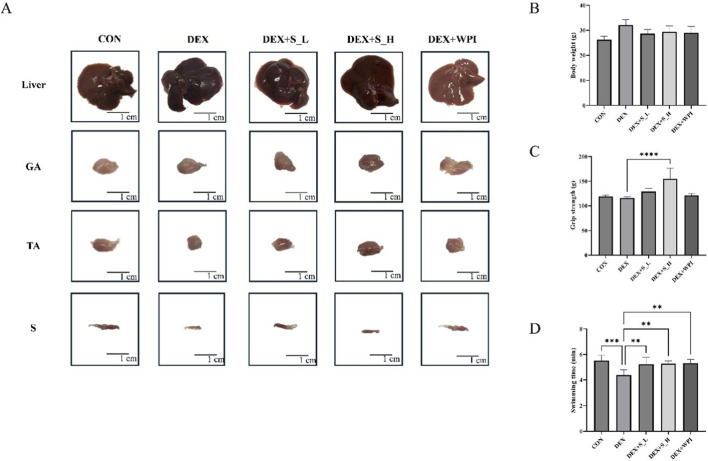
Effect of fermented Soylagen on skeletal muscle mass and muscle performance in dexamethasone (DEX)-induced mice. **(A)** Morphological features of liver, GA, TA and S. **(B)** Body weight, **(C)** Grip strength and **(D)** Swimming time of DEX-induced mice treated with fermented Soylagen. DEX-induced mice were treated with 400 mg/kg and 800 mg/kg fermented Soylagen along with 800 mg/kg whey protein isolates (WPI) for 36 days. After the treatment period grip strength and swimming time were measured to evaluate the muscle performance. Then mice were sacrificed. CON: control group; DEX: dexamethasone injected group; DEX + S_L: dexamethasone injected group treated with low concentration (400 mg/kg) of fermented Soylagen; DEX + S_H: dexamethasone injected group treated with high concentration (800 mg/kg) of fermented Soylagen; DEX + WPI: dexamethasone injected group treated with WPI (800 mg/kg). The results expressed as mean ± SD and the significant values are denoted based on Scheffe’s multiple comparisons test. **p < 0.01, ***p < 0.001 and ****p < 0.0001 compared to DEX group.

To assess the impact of fermented Soylagen on skeletal muscle mass and performance, DEX-induced mice were administered varying concentrations of fermented Soylagen (400 and 800 mg/kg) and WPI (800 mg/kg) for 36 days. Figure 6A shows the morphological characteristics of the liver, GA, TA, and soleus. As shown in the figure, DEX treatment significantly reduced the size of the TA and soleus muscles. However, treatment with fermented Soylagen and WPI effectively reversed the size reduction induced by DEX. Figure 6B shows the average body weight of different treatment groups and displayed no significant difference among the different treatment groups. The current study next evaluates muscle performance by measuring grip strength and swimming time, with the results presented in Figures 6C,D. As shown in the figure, treatment with fermented Soylagen increased grip strength in a dose-dependent manner. Furthermore, the higher dose of fermented Soylagen significantly improved grip strength compared to the DEX treatment (p < 0.05). A similar trend was observed for swimming time, where DEX treatment significantly reduced swimming time (p < 0.01), while administration of fermented Soylagen and WPI significantly increased the swimming time that was decreased by DEX (p < 0.05). Additionally, impact of fermented Soylagen administration on serum ALT and AST levels in DEX-induced mice was evaluated using commercially available kits, with the results shown in Supplementary Figure S2. These findings suggest that the administration of fermented Soylagen does not have any adverse effects on liver health.

### 3.5 Effect of fermented Soylagen administration on protein expression levels of PI3K/Akt/mTOR pathway

**FIGURE 7:**
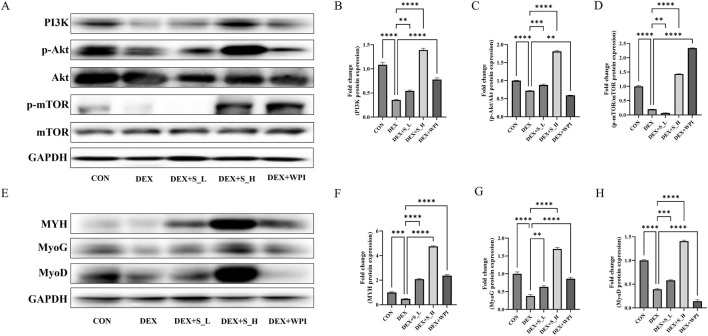
Effect of fermented Soylagen on protein expression levels in gastrocnemius muscle. **(A)** Representative Western blot images of protein expression levels of PI3K, p-Akt, Akt, p-mTOR and mTOR. Densitometry quantification of the protein expression of **(B)** PI3K, **(C)** p-Akt/Akt and **(D)** p-mTOR/mTOR. **(E)** Representative Western blot images of protein expression levels of MYH, MyoG and MyoD. Densitometry quantification of the protein expression of **(F)** MYH, **(G)** MyoG and **(H)** MyoD. Total proteins were extracted from gastrocnemius muscle tissues and protein expression were measured by Western blot analysis. GAPDH was used as internal control to normalize the intensity of protein bands. CON: control group; DEX: dexamethasone injected group; DEX + S_L: dexamethasone injected group treated with low concentration (400 mg/kg) of fermented Soylagen; DEX + S_H: dexamethasone injected group treated with high concentration (800 mg/kg) of fermented Soylagen; DEX + WPI: dexamethasone injected group treated with WPI (800 mg/kg). The results expressed as mean ± SD and the significant values are denoted based on Scheffe’s multiple comparisons test. *p < 0.05, **p < 0.01, ***p < 0.001 and ****p < 0.0001 compared to DEX group.

To determine the effect fermented Soylagen on muscle protein synthesis through the PI3K/Akt/mTOR, the current study employed DEX-induced mice model and protein expression levels were measured after oral administration of fermented Soylagen and WPI for 36 days. Effect of fermented Soylagen on protein expressions levels of PI3K, p-Akt and p-mTOR were shown in Figure 7A. As shown in Figure 7B, the protein expression levels of PI3K were significantly reduced in the DEX group. However, oral administration of fermented Soylagen increased PI3K expression in a dose-dependent manner, with the higher concentration of fermented Soylagen being more effective than WPI and the control. A similar pattern was observed for the protein expression levels of p-Akt, where the reduction in p-Akt expression induced by DEX was upregulated by oral administration of fermented Soylagen. Figure 7C illustrates the protein expression levels of p-mTOR in DEX-induced mice treated with fermented Soylagen and WPI. As shown in the figure, the protein expression of p-mTOR was significantly reduced in the DEX group. However, administration of fermented Soylagen and WPI effectively upregulated the decreased p-mTOR expression caused by DEX. These results suggest that the beneficial effects of fermented Soylagen on sarcopenia may be mediated through the activation of the PI3K/Akt/mTOR signaling pathway.

### 3.6 Effect of fermented Soylagen administration on protein expression levels of MYH, MyoG, and MyoD

MYH, MyoG, and MyoD are essential for muscle development and differentiation, and are therefore believed to play a critical role in muscle atrophy and sarcopenia. In light of this, the current study investigated the protein expression levels of MYH, MyoG, and MyoD following treatment with fermented Soylagen and WPI, with the results presented in Figure 6E. As shown in the figure, DEX treatment significantly reduced the protein expression levels of MYH, MyoG, and MyoD (Figures 7F–H). However, oral administration of fermented Soylagen upregulated these protein expression levels where higher dose showed more significant expression levels than the lower dose. Similarly, treatment with WPI also reversed the DEX-induced reduction in MYH and MyoG resulting in their upregulation.

### 3.7 Effect of fermented Soylagen on histological characteristics of muscle

**FIGURE 8:**
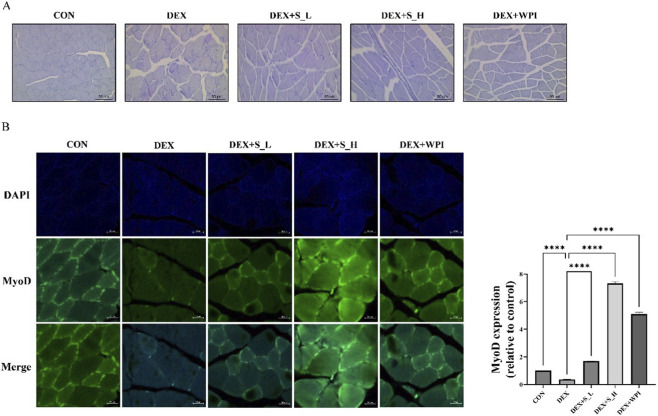
Effects of fermented Soylagen on the histological changes in the skeletal muscle tissues in dexamethasone (DEX)-induced mice. Cross sections of gastrocnemius tissues were stained with H&E stain captured at ×200 magnification **(A)** Representative pictures of tissue staining with H&E. **(B)** Immunofluorescence images of gastrocnemius tissues. The panels represent the fluorescence images of MyoD (green), DAPI (blue) and merged signal. CON: control group; DEX: dexamethasone injected group; DEX + S_L: dexamethasone injected group treated with low concentration (400 mg/kg) of fermented Soylagen; DEX + S_H: dexamethasone injected group treated with high concentration (800 mg/kg) of fermented Soylagen; DEX + WPI: dexamethasone injected group treated with WPI (800 mg/kg). Data are expressed as the mean ± SD. ****p < 0.0001 compared with DEX group.

To assess the effect of fermented Soylagen on the histological characteristics of muscle, the current study utilized a DEX-induced muscle atrophy mouse model and examined the muscle’s histological features *in vivo* through H&E staining. The results are presented in Figure 8A. A key histological characteristic of skeletal muscle atrophy is the reduction in muscle fiber diameter, along with areas where the muscle fibers are loosely arranged (Ko et al., 2020). Considering the association between muscle atrophy and sarcopenia (Nishikawa et al., 2021), the current study aimed to investigate these features in gastrocnemius tissue using H&E staining. The H&E staining of gastrocnemius tissue in the control group revealed normal histology. In contrast, DEX treatment induced pathological changes in the cell structure of the gastrocnemius tissue, characterized by larger intercellular gaps. Moreover, as shown in Figure 8A, the muscle fibers were more widely separated, leaving considerable space between the muscle bundles in the DEX group compared to the control group. However, treatment with fermented Soylagen and WPI gradually improved these pathological changes by narrowing the intercellular gaps. Additionally, the cross-sectional area (CSA) of the muscle fibers was reduced with DEX treatment compared to control group, whereas fermented Soylagen and WPI treatment alleviated this reduction in CSA.

### 3.8 Effect of fermented Soylagen on expression level of MyoD in muscle tissue

To further confirm the effect of fermented Soylagen on muscle atrophy, the protein expression levels of MyoD in different treatment groups were assessed with immunofluorescence staining and the results are shown in Figure 8B. As revealed by the figure the expression level of MyoD was downregulated by the treatment of DEX compared to the control group. Interestingly, oral administration of fermented Soylagen upregulated the expression level of MyoD in a dose dependent manner. Similarly, WPI treatment also contributed to an increase in the DEX-induced reduction in MyoD expression. The Western blot analysis results of the current study also confirm these findings where the protein expression levels of MyoD decreased in the DEX group compared to the control group, while oral administration of fermented Soylagen increased the expression level of MyoD in a dose dependent manner (Figure 7E). Consequently, the findings of the current study suggest the beneficial effect of fermented Soylagen on muscle atrophy, suggesting its potential utilization in future treatments for sarcopenia.

## 4 Discussion

Progressive loss of muscle mass, function, and strength are characteristic features of muscle atrophy and can lead to sarcopenia. Due to the risk factors associated with sarcopenia, there is growing interest in exploring effective preventive and treatment strategies. In this context, dietary and nutritional interventions have been identified as promising strategies to enhance muscle mass, which helps maintain physical performance and function (Walker et al., 2011). Subsequently, recent studies have shown that protein supplementation significantly helps prevent muscle atrophy, as proteins, composed of amino acids, assist in the muscle protein anabolic response, which is conditioned by the availability of branched-chain amino acids (BCAAs) (Liu et al., 2024). Therefore, the potential role of BCAAs in the development and progression of sarcopenia has become a hot spot in research. These BCAAs, which primarily include isoleucine, leucine, and valine, are essential amino acids obtained from dietary sources. They serve as substrates for protein synthesis and act as regulators of muscle protein turnover, thereby playing a key role in muscle protein metabolism (Bai et al., 2022). Recent evidence indicates that disruptions in BCAA metabolism play a role in the pathogenesis of muscle atrophy. Research has shown that individuals with sarcopenia have lower circulating levels of BCAAs compared to healthy individuals (Bai et al., 2022; Kitajima et al., 2018). Therefore, recent literature suggests an association between BCAAs and the prevention of sarcopenia mediated by muscle atrophy.

To demonstrate the beneficial role of BCAAs in sarcopenia, the GO enrichment analysis results of the current study identified branched-chain amino acid biosynthesis as a key biological process and cellular component associated with the core targets of Soylagen in muscle atrophy. Therefore, the potential mechanism of Soylagen in preventing sarcopenia may involve its participation in the branched-chain amino acid biosynthesis process. Additionally, KEGG enrichment analysis identified valine, leucine, and isoleucine biosynthesis as key pathway terms associated with the core targets of Soylagen in sarcopenia, further supporting the potential of Soylagen in preventing sarcopenia through its involvement in BCAA metabolism. Since BCAAs was detected during the amino acid profile analysis of Soylagen, the beneficial effects of Soylagen on sarcopenia may be attributed to the presence of these BCAAs, which play a crucial role in muscle protein synthesis and muscle maintenance.

The disruption of redox balance and the activation of innate immunity are believed to be crucial factors in the development of sarcopenia (Dami et al., 2019). A critical factor in the pathophysiology of sarcopenia is the elevation of reactive oxygen and nitrogen species (ROS/RNS), coupled with a reduction in enzymatic antioxidant defenses, which contributes to oxidative stress (Ardite et al., 2004). Therefore, several recent studies propose antioxidant dietary supplements as a promising strategy to combat age-related declines in muscle mass and performance. Glutathione (GSH) plays a crucial role in maintaining cellular oxidoreductive balance and redox homeostasis/signaling. Research has also highlighted the effect of GSH depletion on the conversion of murine skeletal muscle C2C12 myoblasts into myocytes, a process triggered by the inactivation of growth factors. Thus, GSH is essential for the formation of myotubes from satellite myoblasts by promoting the inactivation of NF-κB. Therefore, preserving adequate GSH levels may help restore muscle mass in conditions characterized by chronic inflammation (Oh et al., 2018). To demonstrate the beneficial role of GSH in sarcopenia, the GO enrichment analysis in the current study identified glutathione biosynthesis as one of the key cellular components of Soylagen in the context of sarcopenia. Similarly, the beneficial effects of Soylagen on sarcopenia may be mediated through its association with glutathione biosynthesis.

Muscle loss associated with aging primarily affects fast-twitch myosin heavy chain (MHC) fibers. The proportion of fast-twitch MHC fibers is positively correlated with muscle strength, highlighting the critical role MHC plays in sarcopenia. Higher proportions of MHC I and lower proportions of MHC IIa fibers are inversely associated with muscle strength and quality (Moore et al., 2015). In line with these findings network pharmacology analysis of the current study identified MHC class I peptide loading complex as one of the key components in GO enrichment analysis.

The reduced intake of essential amino acids in diet adversely affects muscle synthesis in older age, a process that is further combined by the decline in muscular protein synthesis during aging. This phenomenon is referred to as “anabolic resistance” in aging. The dysfunction in mRNA translation and the activation of the mTOR pathway are among the key factors contributing to anabolic resistance. Therefore, recent studies suggest increased protein intake as a promising means to counteract this resistance (Cochet et al., 2023). Research has also shown that BCAAs, especially leucine, isoleucine, and valine, have the ability to activate the mTOR pathway (Yoon, 2017). Therefore, the beneficial effects of Soylagen on muscle atrophy are not solely attributed to its mechanism related to BCAAs, but also to the activation of the mTOR pathway, which is recognized as a key regulator of protein synthesis and metabolism regulation. mTOR, a serine/threonine kinase, monitors changes in the environment and intracellular conditions, such as nutrient levels and energy status, and orchestrates a range of cellular activities, including growth, differentiation, autophagy, survival, and metabolism (Yoo et al., 2021). Confirming the effectiveness of mTOR pathway in muscle atrophy, the findings of the current study confirmed that the treatment of Soylagen enhanced the phosphorylation level of mTOR in both *in vitro* and *in vivo* experiments. Additionally, Soylagen treatment also contributed to increasing the phosphorylation level of p-Akt. Akt, a serine/threonine protein kinase, is crucial for cell survival and proliferation. Additionally, Akt signaling in skeletal muscle is predominantly activated in response to resistance training (Han and Choung, 2022). Moreover, activated Akt promotes protein synthesis by phosphorylating the mammalian target of rapamycin complex 1 (mTORC1) (Srivastava et al., 2024). Therefore, increased skeletal muscle mass observed with Soylagen treatment may be exerted through the Akt/mTOR pathway. These *in vitro* and *in vivo* findings align with the network analysis, which identified Akt as one of the key genes in hub gene identification, further confirming the promising effects of Soylagen in muscle atrophy and, consequently, sarcopenia. The results of the current study also demonstrate that Soylagen treatment increased the expression of PI3K. Recent studies have shown that the PI3K/Akt pathway plays a crucial role in regulating the balance between protein synthesis and degradation, directly influencing muscle mass. Therefore, the promising effects of Soylagen on muscle atrophy may be mediated through the activation of the PI3K/Akt/mTOR pathway. Previous studies have also highlighted the importance of the activation of mTOR pathway in the regulation of muscle protein synthesis (Seo et al., 2022).

The prevention and treatment of muscle atrophy should focus on inhibiting muscle degradation while promoting muscle repair. In this context, the differentiation of satellite cells into myoblasts, myocytes, and myofibers is also crucial, as it is essential for muscle regeneration. The myogenic regulatory factor (MRF) transcription factor family, which includes MyoD, MyoG, Myf5, and Mrf4, is essential for coordinating the various stages of skeletal muscle formation (Jheng et al., 2018). The findings of the current study clearly demonstrate that Soylagen treatment is effective in enhancing the expression levels of muscle regeneration markers MyoD, MyoG, and MyHC. MyoD is considered an early myogenic regulatory factor (MRF), while MyoG is a late differentiation MRF. Therefore, the expression levels of MyoD, MyoG, and MyHC were used to monitor myogenesis as previous studies have identified these as primary markers of sarcopenia. Additionally, Akt activation promotes the expression of muscle-specific proteins and stimulates muscle differentiation (Danboyi et al., 2022).

The magnitude of WPI effects between Western blot and immunofluorescence analyses were not consistent, which may arise from methodological variations. While Western blotting provides quantitative information on total protein expression, immunofluorescence reflects spatial distribution and relative intensity within tissue sections. Therefore, minor discrepancies between these methods are expected and do not necessarily indicate contradictory biological effects.

A limitation of this study is that the effects of fermented Soylagen were not directly compared with its non-fermented counterpart in terms of cell viability. Therefore, the specific contribution of fermentation to the observed bioactivity cannot be conclusively determined. Future studies should include such comparisons to better explain the role of fermentation-derived modifications.

During the study, *in vitro* experiments were conducted using intact Soylagen, which does not fully reflect the physiological conditions following oral ingestion. *In vivo*, dietary protein undergoes enzymatic digestion in the gastrointestinal tract, resulting in peptides and amino acids that are subsequently absorbed by enterocytes. Therefore, the bioactive components of Soylagen present in circulation may differ from those directly applied to cells *in vitro*. Future studies incorporating simulated gastrointestinal digestion models or bioavailability assessments are necessary to better elucidate the physiological relevance of these findings.

Another limitation of the current study is the use of a relatively low dose of DEX, which may have resulted in a less significant reduction in grip strength in DEX-induced mice compared to the control group. This may be attributed to the lower dose of DEX used in the current study compared to previously reported studies (Jheng et al., 2018). Since DEX administration has been reported to cause adverse and severe effects even at low doses over prolonged periods (Lynch et al., 2020), this study used a comparatively lower dose to induce sarcopenia. However, the adverse effects of DEX at different doses, along with its impact on muscle performance, need to be explored in future research to determine the optimal DEX dosage for inducing sarcopenia while minimizing adverse effects.

The current study compared the efficacy of Soylagen on muscle function against whey protein isolates. The findings suggest that Soylagen exhibited similar effects to whey protein isolates while demonstrating enhanced effects on certain parameters. A recent study further supports this, indicating that plant proteins like soy protein can enhance strength and muscle development similarly to whey protein when consumed in adequate amounts to meet leucine requirements (Wróblewska et al., 2018). Another study indicated that whey protein is effective for rapid skeletal muscle growth, while soy protein is more beneficial for weight loss, highlighting the distinct roles each plays in enhancing muscle function (40). However, the effects of Soylagen on muscle function enhancement have been less reported compared to whey protein, as Soylagen is a relatively novel material that requires further research to confirm its impact on muscle function.

## 5 Conclusion

The findings of the current study show that fermented Soylagen treatment is more effective in preventing muscle atrophy compared to unfermented Soylagen. The potential mechanisms underlying the increase in skeletal muscle mass and performance with fermented Soylagen treatment in the *in vivo* model are mediated through the activation of the PI3K/Akt/mTOR pathway. Furthermore, network pharmacology analysis revealed that the beneficial effects of Soylagen on sarcopenia, mediated by muscle atrophy, are primarily linked to the biosynthesis of branched-chain amino acids.

## Data Availability

The original contributions presented in the study are included in the article/Supplementary Material, further inquiries can be directed to the corresponding authors.
