# Supplementary material for: Effect of fermented soy protein isolates containing collagen (Soylagen) on muscle atrophy: insight from network pharmacology analysis and experimental evidence
Source: Front Pharmacol. 2026 Jul 14;17:1836847. doi: 10.3389/fphar.2026.1836847 (PMC13408398; doi:10.3389/fphar.2026.1836847)
Supplement: Supplementary file 2 [file Table1.docx]

| **Pk #** | **RT** | **Name** | **Height** | **Area** | **ESTD Conc(umol/L)** | **Conc(ug/L)** |
| --- | --- | --- | --- | --- | --- | --- |
|  |  | P-Ser |  |  | 0.000 BDL | 0.000 |
|  |  | Tau |  |  | 0.000 BDL | 0.000 |
|  |  | PEA |  |  | 0.000 BDL | 0.000 |
|  |  | Urea |  |  | 0.000 BDL | 0.000 |
| 1 | 11.933 | Asp | 92591 | 2356778 | 24061.869 | 3202634.703 |
| 3 | 16.853 | Thr | 55245 | 1743039 | 17229.614 | 2052046.993 |
| 4 | 18.533 | Ser | 4796 | 147797 | 1445.070 | 151876.892 |
| 5 | 21.507 | AspNH2 | 27487 | 1061053 | 16784.279 | 2217203.239 |
| 6 | 23.527 | Glu | 175025 | 8695677 | 84429.692 | 12419607.659 |
| 7 | 24.893 | GluNH2 | 10370 | 382449 | 4278.870 | 625142.920 |
|  |  | Sar |  |  | 0.000 BDL | 0.000 |
| 8 | 37.647 | Gly | 136539 | 4610328 | 45328.564 | 3402815.270 |
| 9 | 39.640 | Ala | 310211 | 10551970 | 103155.900 | 9190159.114 |
| 10 | 41.600 | Cit | 18987 | 612578 | 5614.842 | 983720.259 |
|  |  | a-ABA |  |  | 0.000 BDL | 0.000 |
| 12 | 45.253 | Val | 219818 | 4004768 | 37861.584 | 4433591.508 |
|  |  | Cys |  |  | 0.000 BDL | 0.000 |
| 14 | 48.367 | Met | 42429 | 1195326 | 11431.182 | 1705532.344 |
|  |  | Cysthi |  |  | 0.000 BDL | 0.000 |
| 15 | 51.887 | Ile | 81166 | 3309788 | 31759.668 | 4166868.467 |
| 16 | 53.620 | Leu | 219256 | 9127631 | 87572.523 | 11489515.047 |
| 17 | 56.107 | Tyr | 6010 | 264763 | 2657.497 | 481538.442 |
| 18 | 59.400 | Phe | 91789 | 3677551 | 37511.353 | 6196875.590 |
|  |  | b-Ala |  |  | 0.000 BDL | 0.000 |
|  |  | b-AiBA |  |  | 0.000 BDL | 0.000 |
| 19 | 68.273 | g-ABA | 3460156 | 114010345 | 1076646.351 | 111002238.779 |
| 20 | 71.427 | Trp | 4353 | 202672 | 3484.879 | 711612.228 |
|  |  | EOHNH2 |  |  | 0.000 BDL | 0.000 |
| 21 | 78.500 | NH3 | 62798 | 7064218 | 74030.857 | 1260745.497 |
|  |  | Hylys |  |  | 0.000 BDL | 0.000 |
| 22 | 90.620 | Orn | 9645 | 336421 | 2746.509 | 363088.428 |
| 23 | 93.660 | Lys | 85660 | 3486977 | 31005.637 | 4533024.165 |
|  |  | 1Mehis |  |  | 0.000 BDL | 0.000 |
| 24 | 98.820 | His | 13335 | 847947 | 8066.903 | 1251983.334 |
|  |  | 3Mehis |  |  | 0.000 BDL | 0.000 |
|  |  | Ans |  |  | 0.000 BDL | 0.000 |
|  |  | Car |  |  | 0.000 BDL | 0.000 |
|  |  | Arg |  |  | 0.000 BDL | 0.000 |

Table S1: Amino acid profile of Soylagen
