# Supplementary figures and images for: Effect of fermented soy protein isolates containing collagen (Soylagen) on muscle atrophy: insight from network pharmacology analysis and experimental evidence
Source: Front Pharmacol. 2026 Jul 14;17:1836847. doi: 10.3389/fphar.2026.1836847 (PMC13408398; doi:10.3389/fphar.2026.1836847)

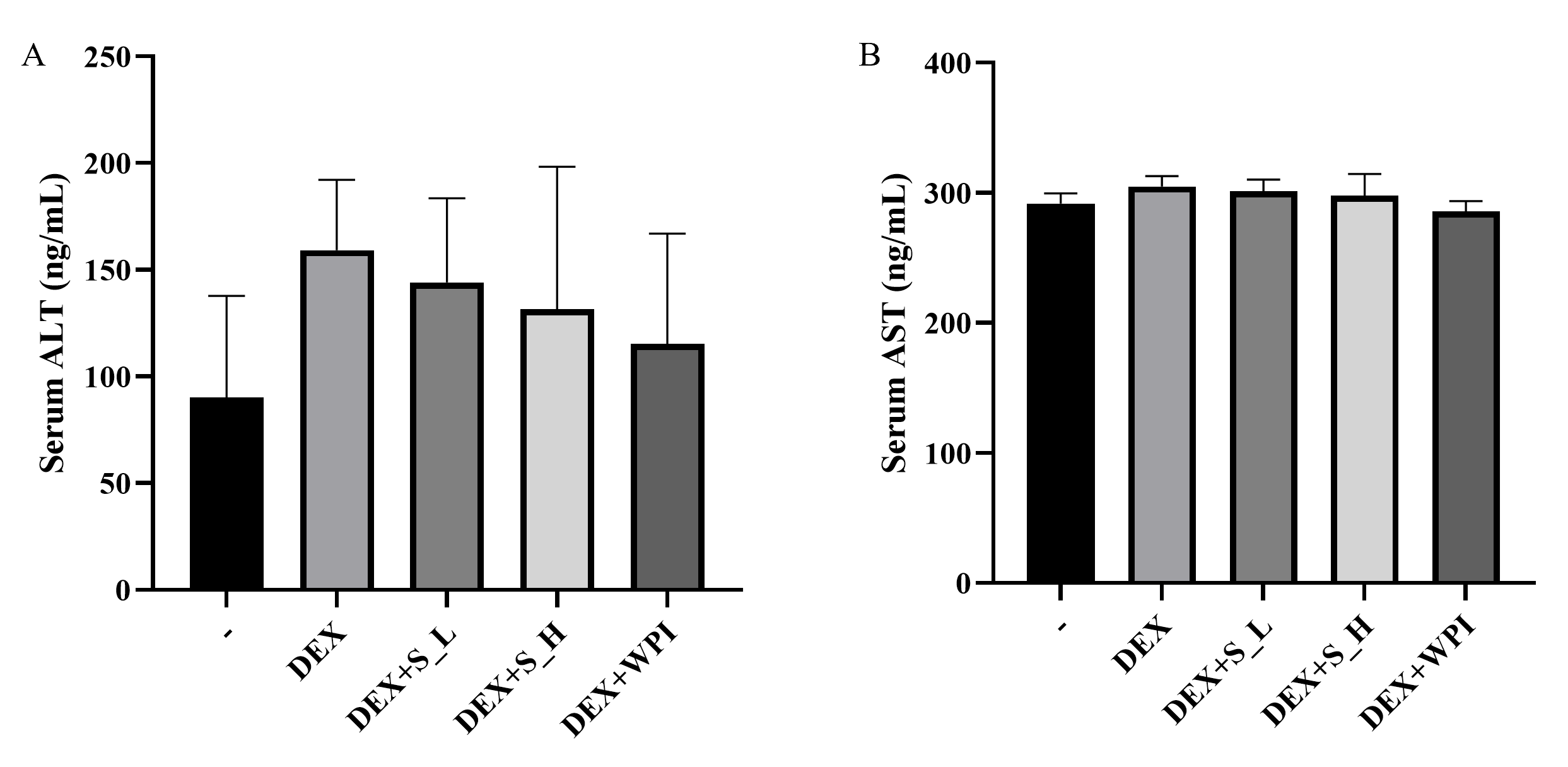

Supplement: Supplementary file 3 [file Image2.tif]

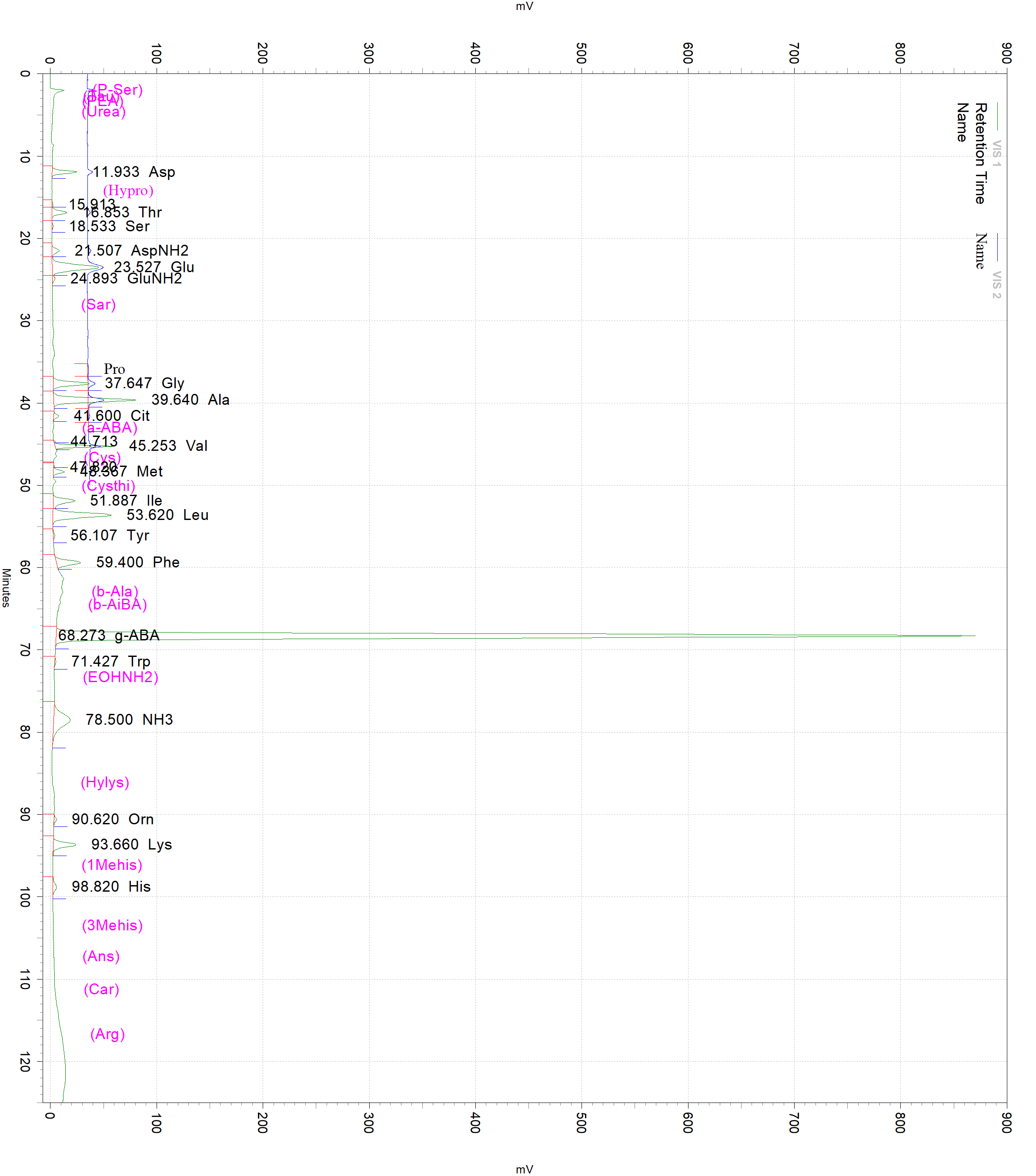

Supplement: Supplementary file 4 [file Image1.png]
